# Decreased INPP5B expression predicts poor prognosis in lung adenocarcinoma

**DOI:** 10.1186/s12935-022-02609-8

**Published:** 2022-05-14

**Authors:** Jun Deng, Xu Lin, Qi Li, Xiao-yu Cai, Lin-wen Wu, Wei Wang, Bo Zhang, Yang-ling Li, Jian Hu, Neng-ming Lin

**Affiliations:** 1 Key Laboratory of Clinical Cancer Pharmacology and Toxicology Research of Zhejiang Province, Affiliated Hangzhou First People’s Hospital, Zhejiang University School of Medicine, Hangzhou, 310006 China; 2 Department of Thoracic Surgery, The First Affiliated Hospital, Zhejiang University School of Medicine, Hangzhou, 310003 China; 3 Department of Clinical Pharmacy, Affiliated Hangzhou First People’s Hospital, Zhejiang University School of Medicine, Hangzhou, 310006 China; 4 Department of Pathology, Affiliated Hangzhou First People’s Hospital, Zhejiang University School of Medicine, Hangzhou, 310006 China; 5 Westlake Laboratory of Life Sciences and Biomedicine of Zhejiang Province, Hangzhou, 310024 China; 6 Cancer Center, Zhejiang University, Hangzhou, 310058 China

**Keywords:** Inositol Polyphosphate-5-Phosphatase B, Prognosis, Lung adenocarcinoma, Bioinformatics

## Abstract

**Background:**

Inositol Polyphosphate-5-Phosphatase B (INPP5B), a inositol 5-phosphatase, plays an important role in many biological processes through phosphorylating PI(4,5)P_2_ and/or PI(3,4,5)P_3_ at the 5-position. Nevertheless, little is known about its function and cellular pathways in tumors. This study aims to investigate the potential role of INPP5B as a diagnostic and prognostic biomarker for lung adenocarcinoma (LUAD), as well as its biological functions and molecular mechanisms in LUAD.

**Methods:**

TCGA, GEO, CTPAC, and HPA datasets were used for differential expression analysis and pathological stratification comparison. The prognostic and diagnostic role of INPP5B was determined by Kaplan–Meier curves, univariate and multivariate Cox regression analysis, and receiver operating characteristics (ROC) curve analyses. The potential mechanism of INPP5B was explored through GO, KEGG, and GSEA enrichment analysis, as well as GeneMANIA and STRING protein–protein interaction (PPI) network. PicTar, PITA, and miRmap databases were used for exploring miRNA targeting INPP5B. In molecular biology experiments, immunohistochemical analyses and Western blot analyses were used to determine protein expression. Co-immunoprecipitation assay was used to detect protein–protein interactions. CCK8 assays and colony formation assays were used for the measurement of cell proliferation. Cell cycle was assessed by PI staining with flow cytometry. Cell migration was performed by Transwell assays and wound healing assays.

**Result:**

INPP5B was decreased in LUAD tissues compared with normal adjacent tissues. And the low expression of INPP5B was associated with late-stage pathological features. In addition, INPP5B was found to be a significant independent prognostic and diagnostic factor for LUAD patients. Hsa-miR-582-5p was predicted as a negative regulator of INPP5B mRNA expression. INPP5B was significantly correlated with the expression of PTEN and the activity of PI3K/AKT signaling pathways, as determined by enrichment analysis and PPI network. In vitro experiments partially confirmed the aforementioned findings. INPP5B could interact directly with PTEN. INPP5B overexpression inhibited LUAD cell proliferation and migration while downregulating the AKT pathway.

**Conclusion:**

Our results demonstrated that INPP5B could inhibit the proliferation and metastasis of LUAD cells. It could serve as a novel diagnostic and prognostic biomarker for LUAD patients.

*Trial registration* LUAD tissues and corresponding para-cancerous tissues were collected from 10 different LUAD patients at Hangzhou First People’s Hospital. The Ethics Committee of Hangzhou First People’s Hospital has approved this study. (registration number: IIT-20210907-0031-01; registration date: 2021.09.13)

**Supplementary Information:**

The online version contains supplementary material available at 10.1186/s12935-022-02609-8.

## Introduction

Lung cancer is the leading cause of cancer-related death worldwide, with a disproportionately high incidence in Asia [1]. Lung adenocarcinoma (LUAD) remains the most prevalent type of lung cancer among all histological subtypes, accounting for more than 40% of lung cancer cases [2]. Based on large-scale multi-omics studies of TCGA, LUAD could be further divided into three subtypes: terminal respiratory unit (TRU), proximal-proliferative (PP), and proximal-inflammatory (PI). The TRU subtype is characterized by lower tumor stage and proliferation, as well as better survival outcomes than the other two, while the PP subtype is the most malignant and characterized by rapid cell proliferation. Although there are several therapeutic options for lung cancer, including surgery, chemotherapy, radiotherapy and targeted therapy, the overall prognosis is still far from satisfactory, with a 5-year survival rate about 19% [3–5]. The difficulty in treating LUAD is mostly due to the high heterogeneity of LUAD [6]. Therefore, exploring more efficient biomarkers is crucial for diagnosis, prognosis, and risk assessment of LUAD.

The PI3K/AKT signalling pathway plays a crucial role in LUAD pathogenesis. PI3K mediates AKT activation and modulates its downstream cellular functions via phosphorylating PI(4,5)P_2_ to PI(3,4,5)P_3_ [7]. This process can be reversed by multiple phosphatase through the dephosphorylation at the 3-, 4-, and 5- positions [8]. For instance, PTEN, a well-characterized tumor suppressor gene, is a 3-phosphoinositide phosphatase that dephosphorylates PI(3,4,5)P_3_ to PI(4,5)P_2_ and thus acts as an AKT suppressor [9]. Similarly, 5-phosphatases can dephosphorylate PI(4,5)P_2_ and/or PI(3,4,5)P_3_ at the 5-position except for inositol polyphosphate 5-phosphatase A (INPP5A), suggesting their tumor suppressive functionalities [10]. INPP5D was reported to negatively regulate PI3K-generated signals, and deletion of INPP5D might lead to the disease progression of spontaneous B cell lymphomas [11, 12]. In addition, the down-regulation of INPP5E, INPP5J, and INPP5K was also observed in various cancers, such as gastric cancer, lung adenocarcinoma, and hepatocellular carcinoma [13–16]. Notably, there have been relatively few studies on the role of INPP5B compared with the other 5-phosphatases family members.

INPP5B belongs to the 5-phosphatases type II family and shares a conserved 5-phosphatase central domain with other family members, implying that it may have similar functions as other members [17]. INPP5B-deficient mice did not show the characteristics of Lowe syndrome, while double-knockout mice for INPP5F and INPP5B were embryonically lethal, indicating that they possessed functional redundancy and were capable of substituting for one another in a particular activity [18]. On the one hand, INPP5B can directly hydrolyze PI(3,4,5)P_3_ at an early stages; on the other hand, it can dephosphorylate PI(4,5)P_2_, and hinder the formation of PI(3,4,5)P_3_ [19–21]. However, the functions and regulatory mechanisms of INPP5B in the field of oncology have never been reported.

The purpose of this study is to determine the diagnostic and prognostic role of INPP5B in LUAD, as well as its biological functions and the regulatory activity on the PI3K/AKT signaling pathway via combining bioinformatics and experimentation. Therefore, our results would shed light on a novel functionality of INPP5B in the development and progression of LUAD.

## Materials and methods

### Data acquisition/processing

The UCSC Xena browser (https://xenabrowser.net/) was used to download Level 3 RNA-sequencing data from LUAD patients, which contains gene expression data and corresponding clinical information from The Cancer Genome Atlas (TCGA, v29.0) and Genotype-Tissue Expression Project (GTEx).

GeneChip data of 33 paired samples were downloaded from GSE10072 in the Gene Expression Omnibus (GEO) database (https://www.ncbi.nlm.nih.gov/geo/). This dataset was used to validate the relationship between INPP5B expression and prognosis. Detailed methods were available in Additional file 1: Supplementary Materials and Methods. The statistical data and information were provided in Additional file 2: Source data and Additional file 3: Table S1.

### Patients and clinical samples

LUAD tissues and corresponding para-cancerous tissues were collected from 10 different LUAD patients at Hangzhou First People’s Hospital. The Ethics Committee of Hangzhou First People’s Hospital has approved this study. The statistics and details were provided in Additional file 3: Table S2.

### Cell culture

Human lung carcinoma cells (A549, H358, H838, PC9) were cultured in 90% RPMI-1640 medium (HyClone, USA) with 10% with 10% (v/v) fetal bovine serum (HyClone, USA). Human lung bronchus epithelial cells (BEAS-2B) and HEK293 cells were cultured in a medium containing 90% DMEM (HyClone, USA) and 10% fetal bovine serum. The cells were cultured at 37 °C in a humidified atmosphere composed of 95% air and 5% CO_2_. All cell lines were obtained from the Shanghai Institute of biochemistry and cell biology (Shanghai, China) and were validated using short ta Indem repeat DNA profiling and mycoplasma testing.

### Transfection

The full-length human INPP5B gene was cloned using BamHI–EcoRI restriction sites into the eukaryotic HA-tag fusion expression vector pcDNA3.1(-)-HA-tag. Similarly, Flag-N-PTEN was generated by putting the Flag-tag at the N terminus of PTEN. Following that, the recombinant plasmids were extracted from Escherichia coli DH-5α. Transient transfection was performed according to the manufacturer’s instructions using jetPRIME (Polyplus, NY, USA). The empty vector was used as a negative control. The transfection efficiency was tested by Western blotting.

### Western blot analysis

Western blot analysis was performed in accordance with earlier published methods [22]. Briefly, protein was extracted from the cells, and concentration was measured with the Bicinchoninic Acid (BCA) protein assay kit (Beyotime). SDS-PAGE was used to separate samples, and they were electrophoretically transferred to PVDF membranes (BioRad, Hercules, CA, USA). After blocking with 5% BSA in TBST, membranes were incubated overnight at 4 °C with primary antibodies, followed by 1 h at room temperature with secondary antibodies. Prior to chemiluminescence visualization of samples, membranes were washed with TBST.

The primary antibodies were as follows: INPP5B (proteintech, 15141-1-AP, 1:1000), p-AKT^(Ser473)^ (CST, 4060S, 1:1000), Wee1 (CST, 13084S, 1:1000), CDK1 (CST, 9116S, 1:1000), Cyclin B1 (CST, 12231S, 1:1000), p-S6 ^(Ser240/244)^(CST, 5364S, 1:1000), β-Actin (Santa Cruz, sc-47778, 1:1000), PTEN (CST, 9188s, 1:1000 for Western blot; 1:50 for IP).

### Statistical analysis

The Wilcoxon signed-rank test was used to determine the expression of INPP5B. The associations between INPP5B expression and the clinicopathological parameters of the LUAD patients were evaluated using the Wilcoxon signed-rank test or the Kruskal–Wallis test. Paired t-test was used to compare two paired groups, while a two-sample t-test was used for non-paired data. The R programming language (version 3.6.3), GraphPad 8.0, ImageJ 1.48, and Adobe Illustrator CS6 were used to conduct all data analyses. (*p < 0.05, **p < 0.01, and ***p < 0.001).

## Results

### INPP5B was down-regulated in LUAD

**Fig. 1:**
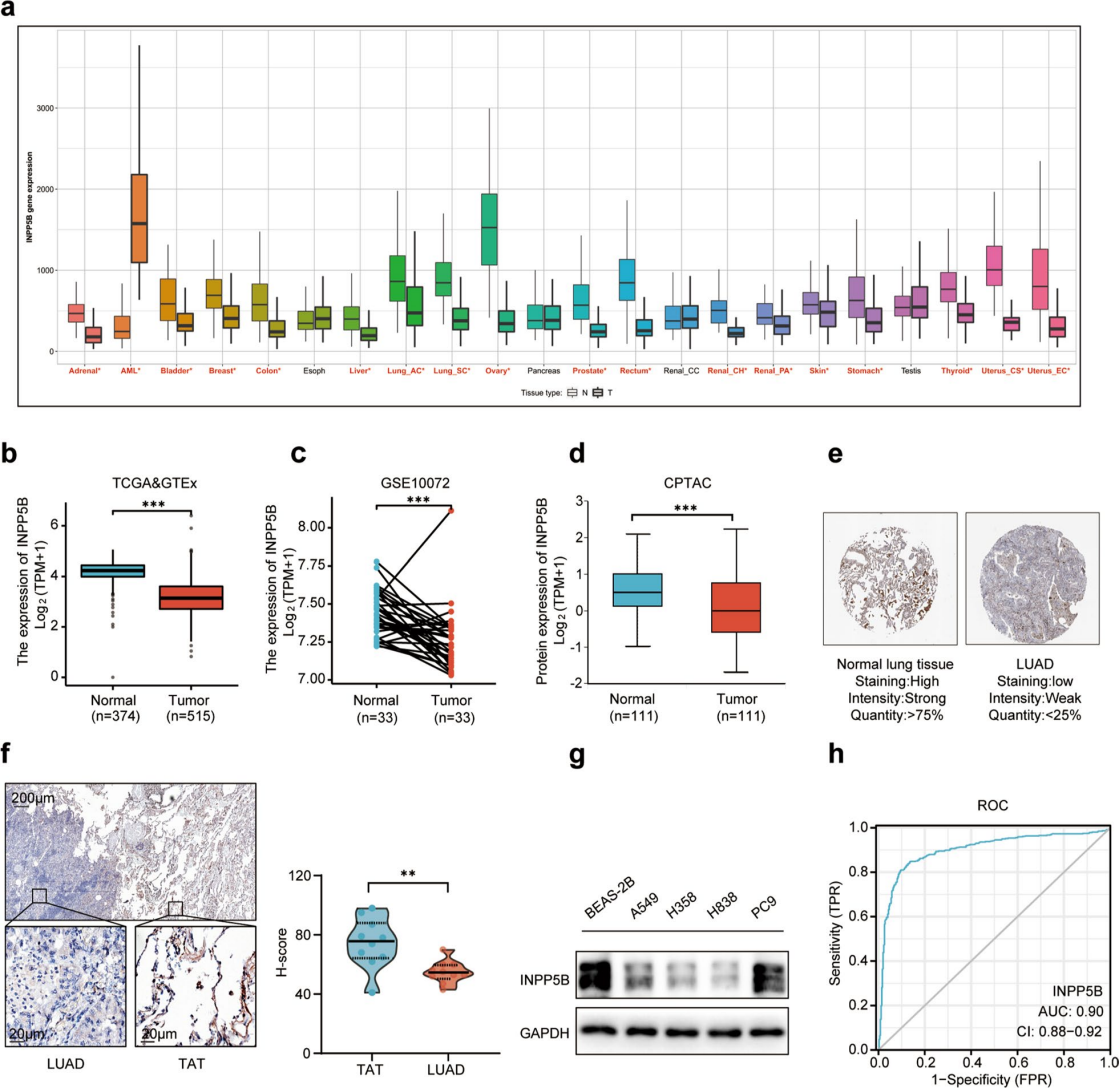
INPP5B expression in LUAD tissues at the mRNA and protein levels. **a** Differential expression of INPP5B in pan-cancer by RNASeq obtained from the TNMplot. Siginificant differences by Mann–Whitney U test marked with red*; black indicated no difference. **b** Expression of INPP5B in LUAD tissues (TCGA, n = 374) and healthy lung tissues (TCGA and GTEx, n = 515) by RNASeq. **c** Paired expression data for LUAD and adjacent normal tissues by RNASeq in GSE10072 dataset (n = 33). **d** The expression level of INPP5B total protein between normal tissue and primary tissue of LUAD based on the CPTAC dataset (n = 111). **e** Immunohistochemistry tissue microarray images of proteins INPP5B obtained from the HPA database. **f** Immunohistochemistry images of proteins INPP5B for LUAD tissue samples and tumor adjacent tissue (TAT). **g** Western blot analysis of INPP5B expression in BEAS-2B cells and four LUAD cell lines. *p < 0.05, **p < 0.01, ***p < 0.001. **h** ROC (Receiver operator characteristic curve) analysis of INPP5B in LUAD patients based on TCGA and GTEX data. AUC area under the curve

Currently, the relationship between INPP5B and oncogenesis is poorly understood. To address this question, we conducted a comprehensive analysis of 22 different types of tumors from TCGA. As a result, the expression of INPP5B was decreased in 17 different types of solid tumors (Fig. 1a). We further assessed the expression of INPP5B in the LUAD samples of TCGA compared with normal controls of TCGA and GTEx. We found that the expression level of INPP5B mRNA was significantly decreased in LUAD tissues (Fig. 1b). This result was further validated in paired samples of GSE10072 (Fig. 1c). The CPTAC database analysis revealed that the INPP5B protein expression was significantly reduced in LUAD tissues compared to normal lung tissues (Fig. 1d). Likewise, IHC on tissue microarray from the HPA database indicated that LUAD tissues had significantly lower levels of INPP5B than normal lung tissues (Fig. 1e). Our experimental data were consistent with the results of bioinformatics analysis. The expression of INPP5B was significantly downregulated in LUAD tissues compared with paired para-cancerous tissues, as determined by IHC (Fig. 1f). Western blot analysis confirmed that INPP5B protein levels were dramatically decreased in LUAD cells (Fig. 1g). In addition, the potential for INPP5B to serve as a diagnostic biomarker for LUAD patients was investigated using Receiver operating characteristic (ROC) curve analysis. ROC curve analysis based on the TCGA database showed that INPP5B appeared to be a promising diagnostic biomarker for patients with the value of area the under curve (AUC) equals to 0.90 (Fig. 1h). ROC curve analysis in GSE10072 data revealed similar results with the AUC value equal to 0.80 (Additional file 4: Fig. S1a). Taken together, these findings indicated that the expression of INPP5B was significantly higher in normal lung tissues than LUAD tissues at both transcriptional and proteomic levels.

### Low expression of INPP5B was associated with late-stage pathological features

**Fig. 2:**
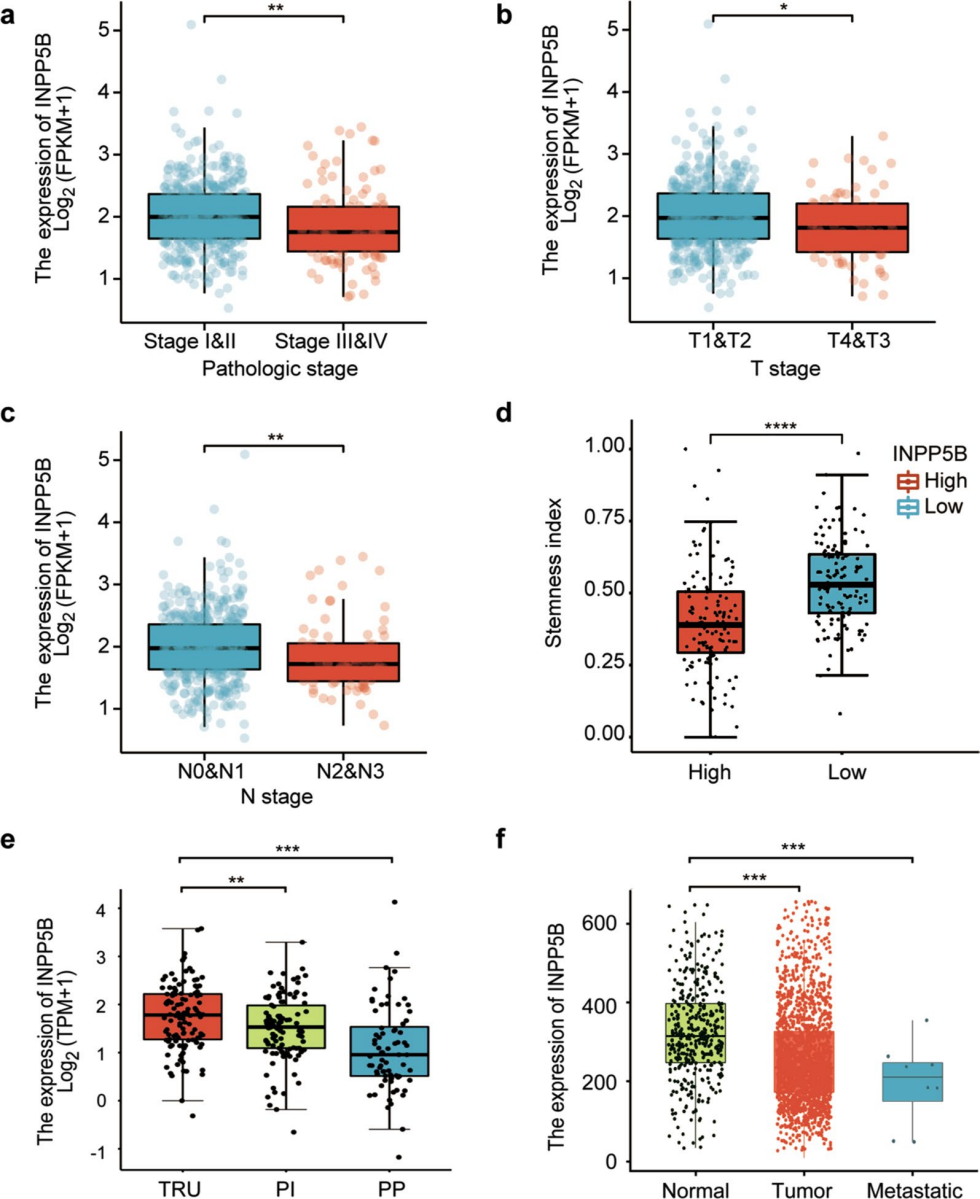
The correlation of INPP5B mRNA expression with risk factors of LUAD patients in TCGA database. **a** Pathologic stage (Stage I & Stage II = 417; Stage III & Stage IV = 110) **b** T stage (T1 & T2 = 464; T3 & T4 = 68) **c** N stage (N0 & N1 = 443, N2 & N3 = 76) **d** Cancer cell stemness (High = 127; Low = 126). **e** LUAD gene expression subtypes included terminal respiratory unit (TRU), proximal proliferative (PP), and proximal Inflammatory (PI). The data were collected from TCGA Portal (http://www.tcgaportal.org/TCGA/Lung_TCGA_LUAD/index.html). **f** Compare Tumor, Normal and Metastasis. The data were collected from Tnmplot (https://tnmplot.com/analysis/). *p < 0.05, **p < 0.01, ***p < 0.001

The TNM staging system is the most common international staging system for malignant tumors. The T-stage refers to the size and metastasis of the primary tumor. The N-stage score is based upon the extent of lymph node involvement, and The M-stage score indicates the extent of distant metastasis [23]. Given the differences in expression of INPP5B between LUAD tissues and adjacent normal tissue, we sought to determine whether the expression of INPP5B was correlated with clinical-pathological parameters of LUAD patients. As shown in Fig. 2, INPP5B mRNA levels was negatively related to the pathological stage (a), T stage (b), and N stage (c) of LUAD patients. Namely, INPP5B expression was down-regulated in advanced stages LUAD compared with early stages LUAD. Meanwhile, LUAD tissues with lower INPP5B expression exhibited higher cancer stemness properties (Fig. 2d; Additional file 1). And the expression of INPP5B in the proximal proliferative (PP) and proximal inflammatory (PI) subtypes were lower than in the terminal respiratory unit (TRU) subtype (Fig. 2e). Furthermore, the metastatic tumor expressed lower levels of INPP5B than the non-metastatic primary tumor or normal lung tissues (Fig. 2f). These results suggested low INPP5B levels might be an unfavorable prognostic factor for LUAD patients.

### Low expression of INPP5B was associated with poor survival of LUAD patients

**Fig. 3:**
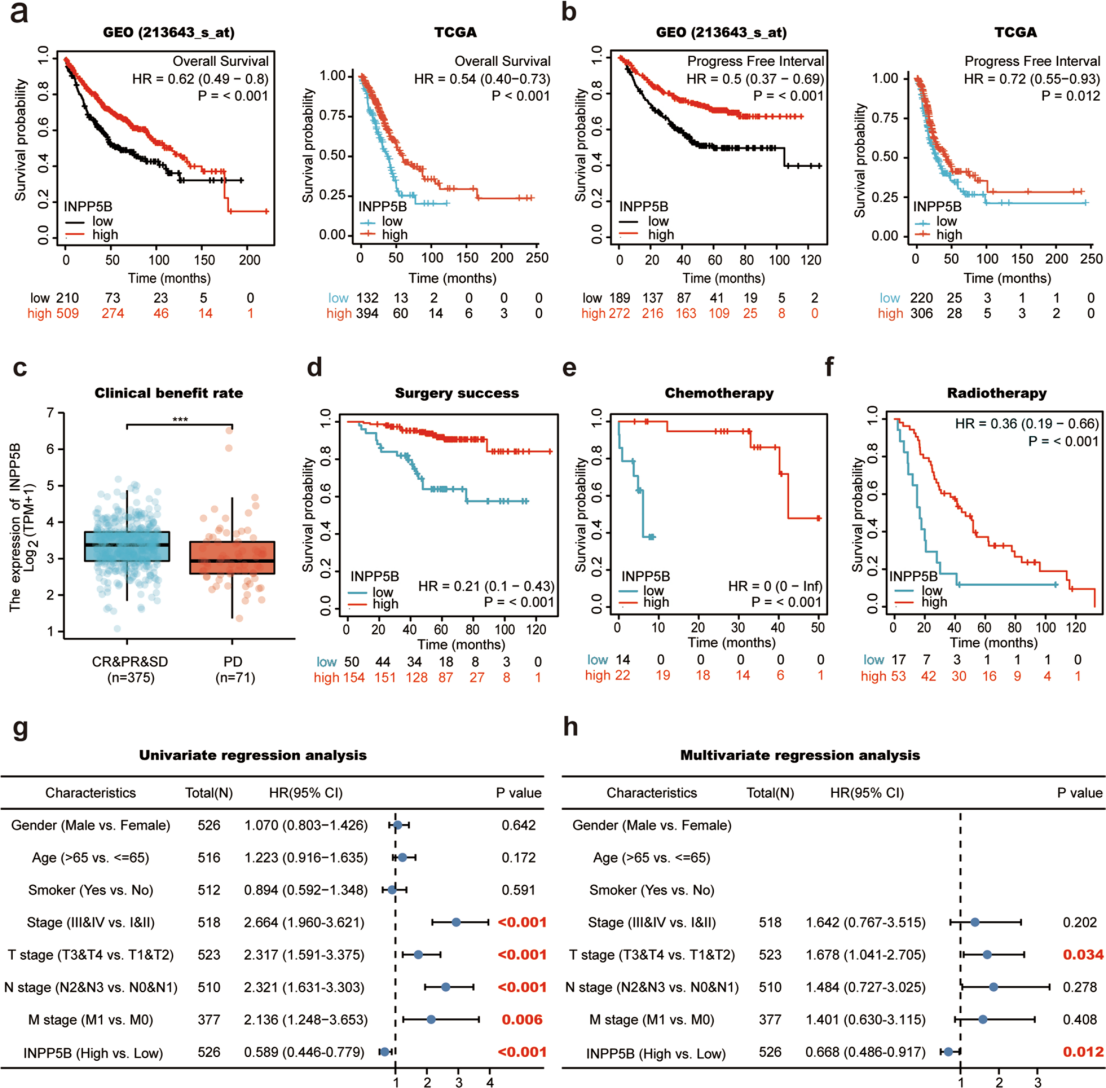
Prognostic role of INPP5B in lung adenocarcinoma. **a**, **b** KM survival curves for overall survival (**a**) and progression free interval (**b**) in LUAD patients from the Kaplan–Meier plotter online database or TCGA database. Grouping by “auto select best cutoff”. **c** Primary therapy outcome included complete response (CR), partial response (PR), stable disease (SD), progressive disease (PD). Clinical benefit rate (CBR) = CR + PR + SD (PR&CR&SD = 375, PD = 71). **d**–**f** Overall survival of patients with lung adenocarcinoma after successful surgery (**d**), Chemotherapy (**e**) and Radiotherapy (**f**), respectively. The data were collected from the Kaplan–Meier plotter online database. Grouping by “auto select best cutoff”. **g**, **h** Univariate analysis (**g**) and Multivariate analysis (**h**) of TCGA database. HR > 1 indicates poorly prognostic; HR < 1 indicates improved OS and any characteristic crossing the line at 1 is not significant

To determine the effect of INPP5B expression on survival rate of LUAD patients, we analyzed the prognostic value of INPP5B expression using TCGA data and GEO data. As demonstrated by the KM curve, there was a strong positive correlation between INPP5B expression and overall survival (OS) period and progression-free interval (PFI) of LUAD patients (Fig. 3a, b). In terms of treatment-related prognosis, LUAD patients with higher expression of INPP5B had a higher clinical benefit rate (Fig. 3c). Besides, LUAD patients with higher INPP5B expression had a higher overall survival rate after surgery (Fig. 3d), chemotherapy (Fig. 3e), or radiotherapy (Fig. 3f). The Univariate Cox analysis revealed that pathologic stage, T stage, M stage, and N stage were all risk factors, whereas INPP5B was a protective factor (HR = 0.589; 95% CI 0.446–0.779; *p* < 0.001) (Fig. 3g). Multivariate Cox analysis showed that INPP5B might be an independent factor of beneficial prognosis of LUAD patients (HR = 0.688; 95% CI 0.478–0.972; *p* = 0.012) (Fig. 3h).

### Enrichment analysis explored INPP5B-related signaling pathways in LUAD

**Fig. 4:**
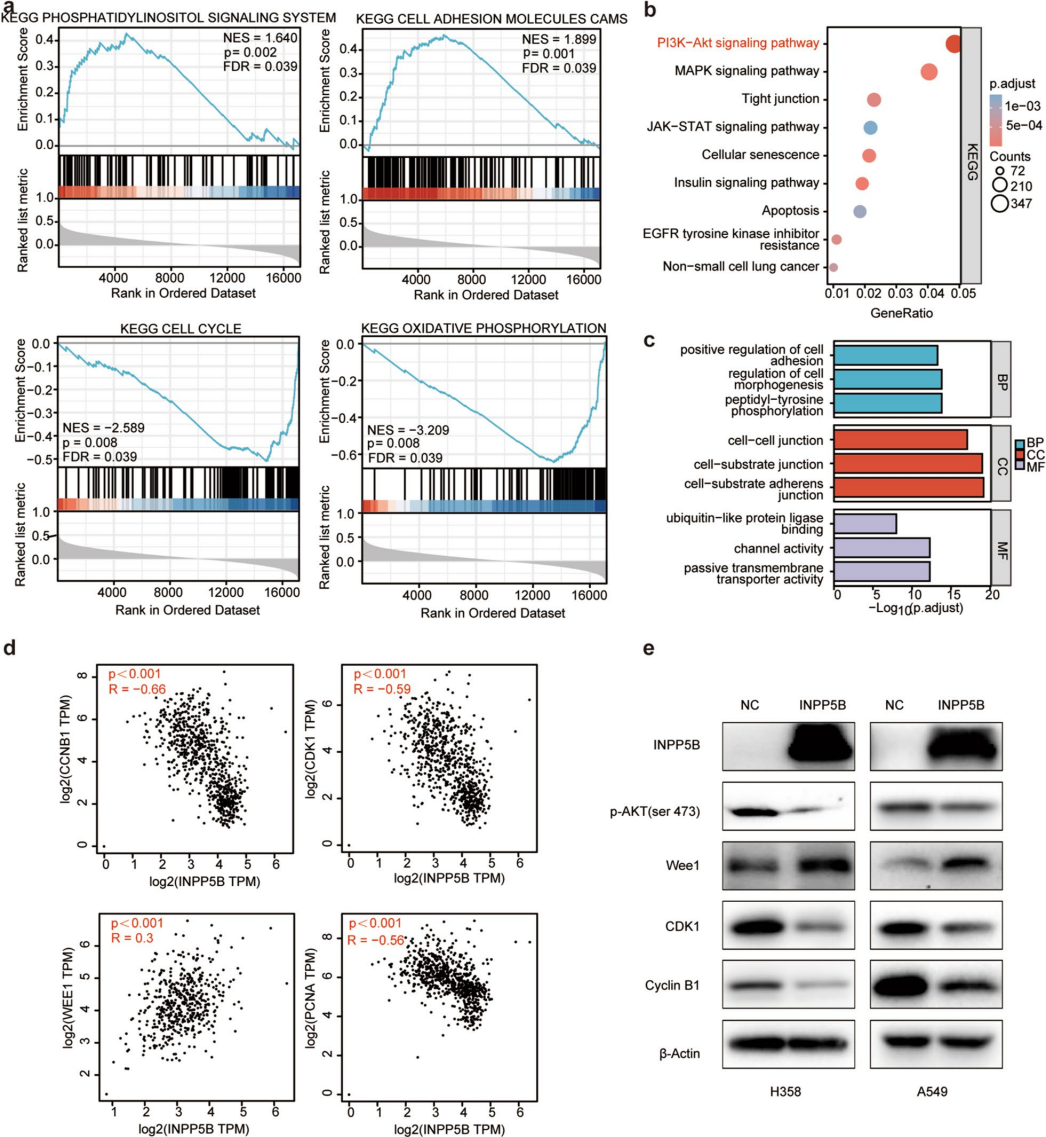
Pathway enrichment analysis of INPP5B. **a** Enrichment plots from GSEA. NES, normalized enrichment score of GSEA. p < 0.05 and FDR < 0.25 were considered statistically significant for GSEA. **b** KEGG signaling pathway enrichment analysis. **c** Gene ontology analysis included biological process (BP), molecular function (MF), and cellular component (CC). **d** The expression relationship between INPP5B expression and cell cycle-related genes. **e** Western blot analysis validation of AKT signaling pathway in LUAD cell lines. Experiments were repeated three times, and data from a representative experiment are shown

To investigate the potential biological functions and molecular mechanisms of INPP5B in regulating LUAD, enrichment analysis was performed by GSEA and GO/KEGG analyses. The GSEA analysis revealed that the terms “phosphoinositol signaling system” and “cell adhesion molecules CAMs” were differentially enriched in LUAD samples expressing high levels of INPP5B. The terms “cell cycle” and “oxidative phosphorylation”, on the other hand, had a strong negative correlation with INPP5B expression (Fig. 4a). In line with GSEA results, the outcomes of GO and KEGG analyses revealed that the co-expressed genes of INPP5B were mainly associated with “positive regulation of cell adhesion”, “cell–cell junction” (Fig. 4c), and “PI3K/AKT signaling pathway” (Fig. 4b). All of these terms indicated that INPP5B exerted a significant effect on tumor proliferation and metastasis, which were consistent with results from Fig. 2. To elucidate this finding, we used GEPIA database to analyze the association between INPP5B expression and various cell cycle-related genes. The results showed that INPP5B was significantly positively correlated with the expression of Wee1, while negatively correlated with CyclinB1, CDK1, and PCNA (Fig. 4d). Combining these results, we speculated that INPP5B might play a role in PI3K/AKT signaling pathway. In our experimental studies, the western blot analysis showed that the overexpression of INPP5B inhibited the activation of AKT signaling pathway, which in turn increased Wee1 protein expression, whereas reduced CDK1 and Cyclin B1 protein levels (Fig. 4e). Taken together, these data indicated that INPP5B might modulate the AKT signaling pathways to influence cell proliferation and migration, thereby limiting the occurrence and progression of LUAD.

### INPP5B interacted with PTEN in LUAD

**Fig. 5:**
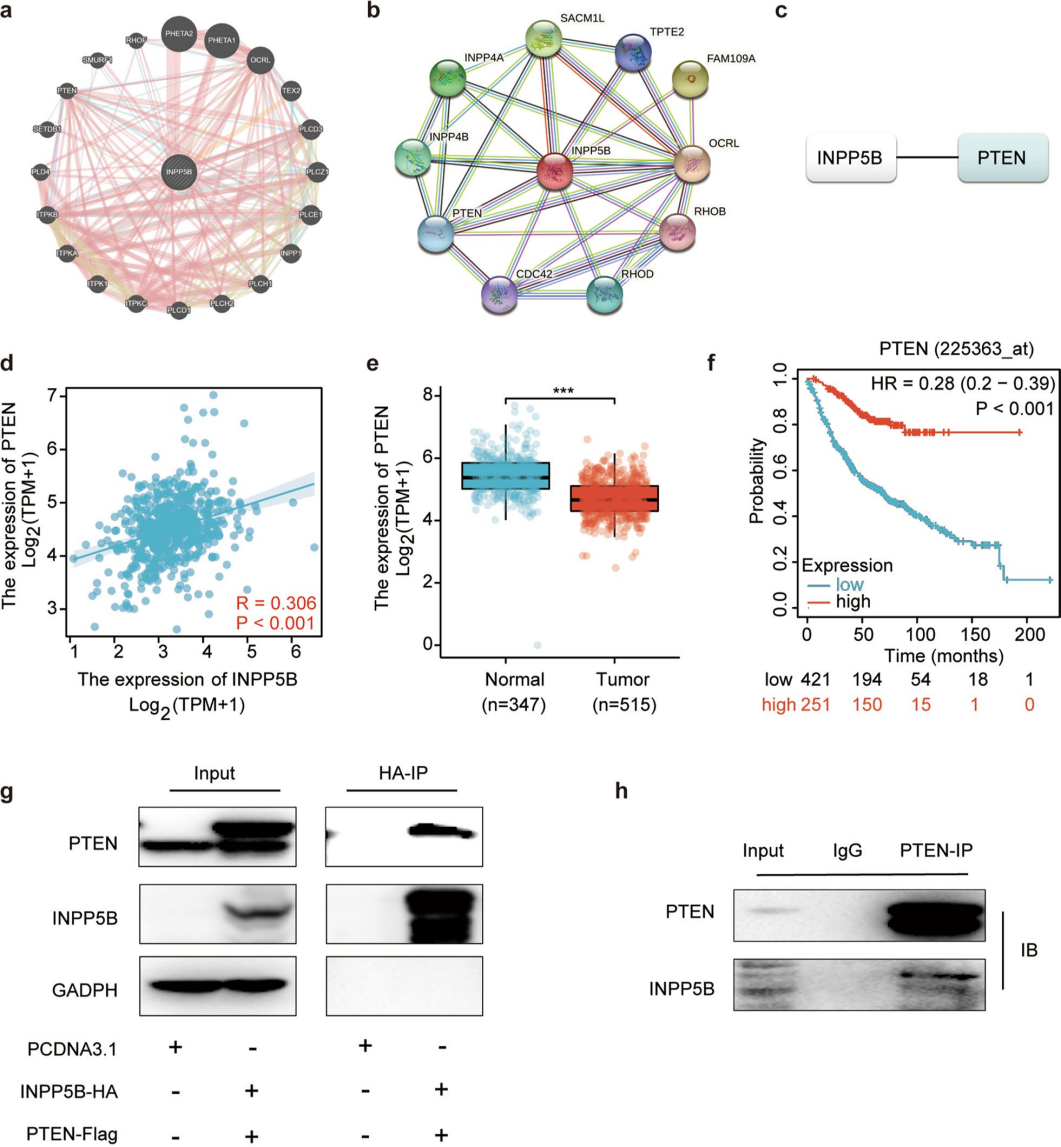
INPP5B-involved protein–protein interaction network. **a**–**c** The INPP5B-involved protein–protein interaction network constructed by STRING database (**a**) and GeneMANIA database (**b**), respectively. The intersections of above datasets identified as key genes (**c**). **d** The expression relationship between INPP5B and PTEN in lung adenocarcinoma from TCGA database. **e** Expression of PTEN in LUAD tissues (TCGA) and healthy lung tissues (TCGA and GTEx) by RNASeq. **f** Survival analysis of PTEN in LUAD determined by Kaplan–Meier plotter database. **g** Co-immunoprecipitation of exogenous INPP5B with exogenous PTEN in HEK293 cells. **h** Co-immunoprecipitation of endogenous PTEN with endogenous INPP5B in A549 cells

In order to investigate the molecular mechanism of INPP5B, we constructed INPP5B-involved PPI networks using GeneMANIA and STRING databases, respectively (Fig. 5a, b). By taking the intersection of results from GeneMANIA (a) and STRING (b), the two most relevant genes were identified as PTEN. We then examined the expression correlations between INPP5B and PTEN using the TCGA database. The results revealed that INPP5B expression was statistically positively associated with PTEN (R = 0.306, *p* < 0.001) (Fig. 5c). The results of the TCGA and GTEx datasets showed lower expression of PTEN in LUAD tissues than that in normal tissues (Fig. 5d). And Kaplan–Meier plotter overall survival (OS) analysis revealed that lower PTEN expression was associated with worse OS in LUAD patients (Fig. 5e). Subsequently, The interactions between both exogenous (Fig. 5g) and endogenous (Fig. 5h) INPP5B with PTEN were confirmed by co-immunoprecipitation (co-IP) in HEK293 and A549 cells.

### Hsa-miR-582-5p upregulation might be responsible for the loss of INPP5B in LUAD patients

**Fig. 6:**
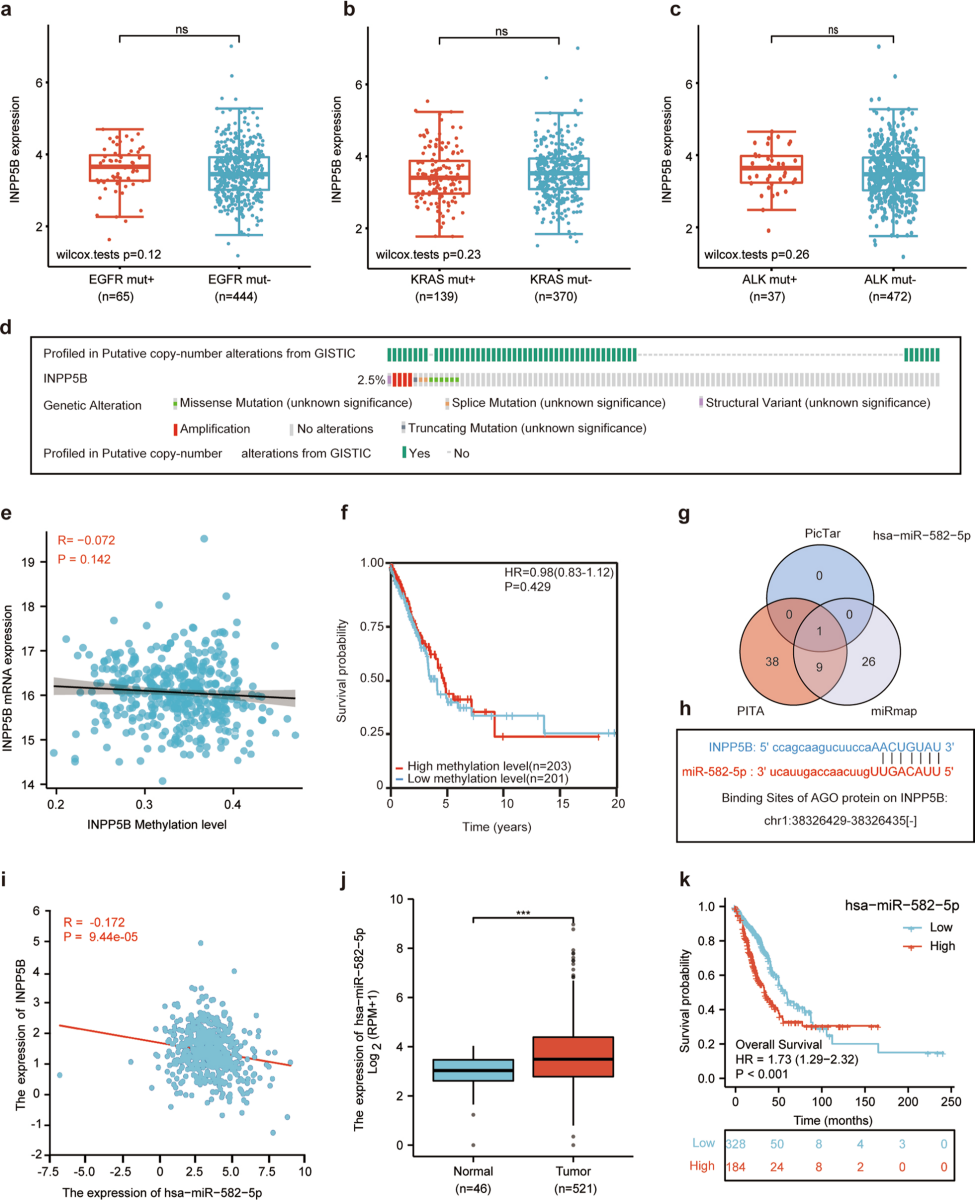
Reasons for the decreased expression of INPP5B in LUAD patients. **a** Correlation of EGFR mutations and INPP5B expression in LUAD tissues. **b** Correlation of KRAS mutations and INPP5B expression in LUAD tissues. **c** Correlation of ALK mutations and INPP5B expression in LUAD tissues. **d** Mutation rate of INPP5B in LUAD patients from the TCGA dataset, n = 566 **e** Correlation of INPP5B expression and INPP5B methylation level in LUAD. **f** Survival analysis of INPP5B methylation level in LUAD patients. **g** The intersection results of PicTar, PITA, and miRmap databases for microRNA targeting INPP5B prediction. **h** Possible binding sites between hsa-miR-582-5p and INPP5B. **i** Correlation between INPP5B expression and hsa-miR-582-5p expression in LUAD tissues. **j** Expression of hsa-miR-582-5p in LUAD patients. **k** Survival analysis of hsa-miR-582-5p in LUAD patients

There are multiple causes for the loss of tumor suppressor genes, the most common of which include genetic mutations and epigenetic alterations [24]. By searching the TCGA and cbioportal databases, we found that the expression of INPP5B was not related to typical driver-gene mutations of LUAD, such as EGFR mutation (Fig. 6a), KRAS mutation (Fig. 6b), and ALK mutation (Fig. 6c). And INPP5B exhibited a low mutation rate in LUAD tissues (2.5%, 14/566) (Fig. 6d). In addition, we found that the expression of INPP5B did not correlate with methylation levels (Fig. 6e, f). microRNAs can be combined with the 3′ untranslated region (UTR) of the target gene to down-regulate the expression level of their target gene. We identified miRNAs that target INPP5B by taking the intersection of results from the PicTar, PITA, and miRmap databases (Fig. 6g). We got hsa-miR-582-5p and predicted its binding site to INPP5B (chr1:38,326,429–38,326,435) (Fig. 6h). The correlation analysis results revealed that the expression of INPP5B and hsa-miR-582-5p was negatively correlated (*p* < 0.001) (Fig. 6i). The result from TCGA indicated that the expression level of hsa-miR-582-5p was increased in LUAD patients (Fig. 6j). And higher hsa-miR-582-5p expression was associated with worse OS (Fig. 6k). Together, these results implied that increased hsa-miR-582-5p might account for the loss of INPP5B rather than genomic alterations or DNA methylation in LUAD patients.

### INPP5B inhibited both proliferation and migration of LUAD cells in vitro

**Fig. 7:**
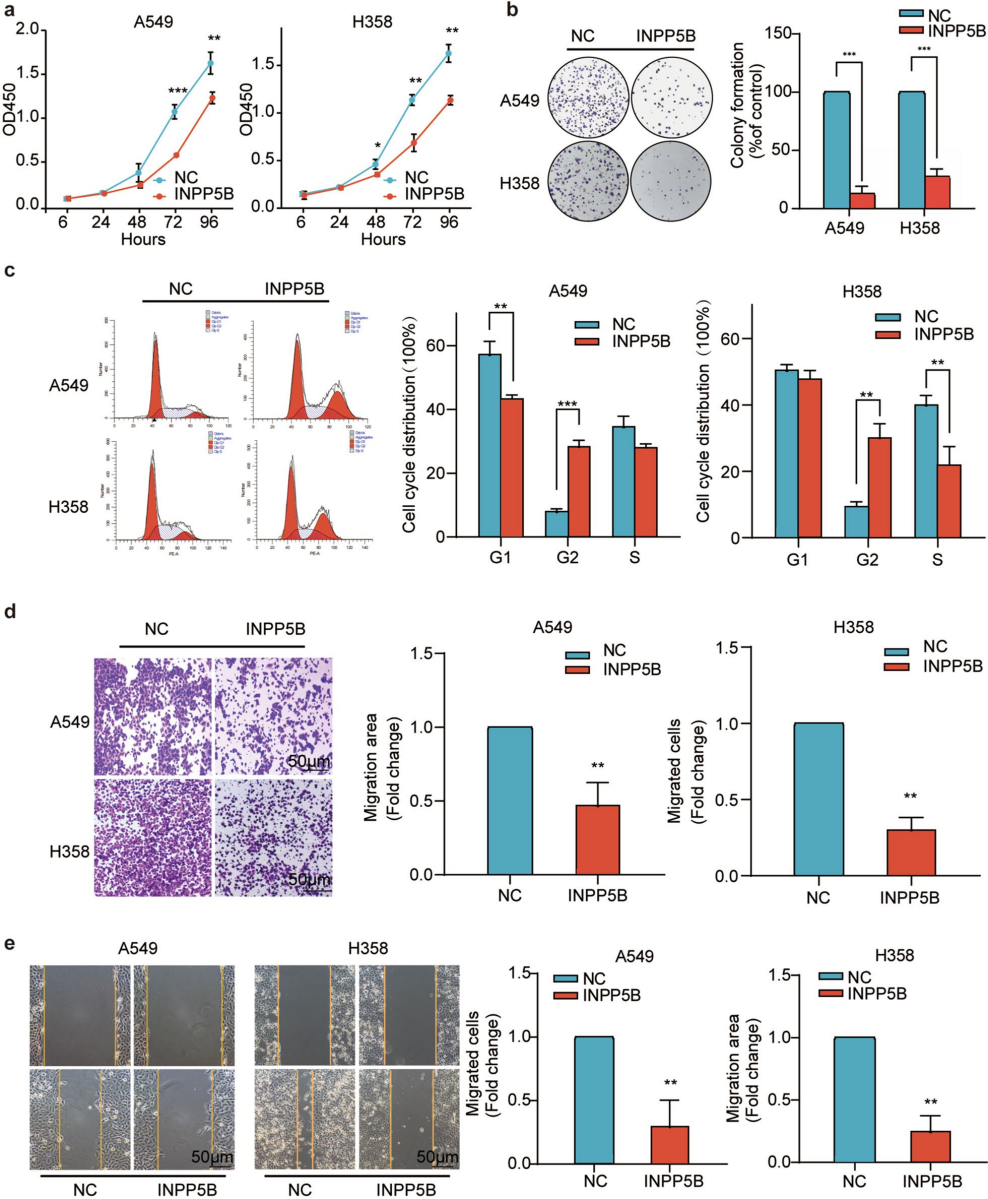
Validation of overexpressed INPP5B as a tumor suppressor in vitro. **a** The CCK-8 assay showed the overexpression of INPP5B inhibited proliferation of lung adenocarcinoma cells. **b** The colony formation assay showed INPP5B overexpression suppressed the proliferation abilities of lung adenocarcinoma cells. **c** Cell cycle and statistical analysis showed significant G2 arrest after INPP5B overexpression. **d** Results of wound healing assay showed that INPP5B overexpression inhibited the migration ability of lung adenocarcinoma cells. Scale bar: 50 μm. **e** Results of transwell assay showed significant decrease in the migration ability of lung adenocarcinoma cells after INPP5B overexpression. Scale bar: 50 μm. *p < 0.05, **p < 0.01, ***p < 0.001

Given that the results of bioinformatics analysis suggested that INPP5B was associated with proliferation and metastasis of LUAD cells, we further verified its biological functions by in vitro experiments. CCK8 assay revealed that the overexpression of INPP5B noticeably inhibited the proliferation of LUAD cells after 48 or 72 h transfection (*p* < 0.01) (Fig. 7a). Colony formation assay revealed a marked reduction of colonies formed by INPP5B overexpressing cells (*p* < 0.001) (Fig. 7b). Flow cytometry analysis demonstrated that the overexpression of INPP5B caused 28.46 ± 1.51% or 30.16 ± 2.53% cells arrested in G2/M phase in A549 and H358 cells, respectively (Fig. 7c). Additionally, INPP5B overexpression significantly impaired both the vertical and horizontal migration abilities of LUAD cells (*p* < 0.01) (Fig. 7d, e). As a result of our findings, INPP5B could inhibit the proliferation and migration of LUAD cells in vitro.

## Discussion

Despite great strides have been made in the understanding of fundamental molecular mechanisms of INPP5B, little is known about its function and cellular pathways in tumors [20]. Here, we demonstrated a causal relationship between decreased INPP5B expression and the development of lung adenocarcinoma (LUAD) by using combinatorial tools consisting of both bioinformatics analysis and experimental studies. In the first place, we discovered that INPP5B expression was decreased in a variety of solid tumors, especially LUAD. And the low expression of INPP5B was associated with late-stage pathological features and poor prognosis in LUAD. These results were consistent with a recent study in which a high mutation rate of INPP5B was observed in the genome-wide mapping of melanocytic neoplasms [25]. Furthermore, changes in the expression of INPP5B have also been found in the metabolomic data of hepatocellular carcinoma [26]. These findings suggested that loss of function or lower functioning of INPP5B might be related to the initiation and progression of tumors.

In view of the differential expression of INPP5B between LUAD and adjacent normal tissue, INPP5B might serve as a potential diagnostic and prognostic indicator in LUAD. This was partially supported by a recent study that was published when we were preparing the current manuscript. Han et al. [27] developed a four-gene signature including INPP5B which could effectively predict the recurrence of early lung cancer patients and associated survival chances following surgery. However, their study was based on a model of multi-gene signatures. And it remained unknown whether INPP5B could be used as an independent diagnostic or prognostic biomarker of LUAD. Besides, their study emphatically demonstrated the post-operative survival impact of the model, but not its role in the development of LUAD. Therefore, the biological function of INPP5B in LUAD even in tumors has not been reported so far. Our study further supplemented the biological function of INPP5B in LUAD. We found that low INPP5B expression was associated with advanced pathological stage and malignant progression in LUAD patients. Interestingly, the differential expression of INPP5B in TMN staging and proliferative subtypes of LUAD, to some extent, reflected that INPP5B might affect tumor proliferation and metastasis. These parts of results were consistent with enrichment analysis data which showed that INPP5B was associated with terms of “molecular adhesion” and “cell cycle functions”. We further validated this hypothesis in vitro. Overexpression of INPP5B significantly inhibited the proliferation and migration of LUAD cells, blocking the cells in G2/M phase. Certainly, further work in vivo was necessary to confirm the anti-proliferation and anti-metastasis functions of INPP5B in LUAD.

Although several inositol phosphatases have been shown to suppress AKT signaling through dephosphorylating phosphoinositides, the function of INPP5B in regulating AKT signaling in tumors has not been directly tested so far. Oomset et al. [28] observed that loss of INPP5J in breast cancer promotes AKT activation, leading to the promotion of tumor growth. Similarly, Bohdanowicz et al. [29] found that APPL1 recruited INPP5F and INPP5B, effectively terminating PI(3,4,5)P_3_ synthesis via substrate depletion and direct dephosphorylation, thereby limiting the duration of AKT activation. Our findings were consistent with the previous studies. GSEA enrichment analysis showed that INPP5B was significantly enriched in PI3K/AKT pathway. Western blot analysis revealed that INPP5B increased the expression of Wee1 by dephosphorylating AKT, inhibiting the formation of CyclinB1-CDK1 complexes, and thereby arresting the cells in the G2/M phase. PTEN is known to be a tumor suppressor by inhibiting the activation of PI3K/AKT pathway [30]. PPI analyses indicated that INPP5B and PTEN might interact. We confirmed this finding through both endogenous and exogenous co-immunoprecipitation experiments. Surprisingly, although INPP5B could interact with PTEN, our experimental results indicated that overexpression of INPP5B had no effect on PTEN expression (Additional file 4: Fig. S1b). We speculated that the interaction between INPP5B and PTEN may be functionally or structurally complementary. Other researchers also shared similar interpretations. Kofuji et al. [31] observed that the dephosphorylation function of INPP4B could act as a “backup” mechanism when PTEN is deficient. It will be of great interest to explore whether INPP5B can also exert its dephosphorylation effect through interacting with PTEN. The investigation now in progress in our laboratory may clarify this question.

After identifying the anti-tumor properties and mechanism of INPP5B, we wanted to explore the underlying cause of the down-expression of INPP5B in LUAD patients. microRNAs (miRNA) are endogenous non-coding RNA that can bind to the target 3′-UTR to inhibit translation or promote transcription degradation of the target gene [32]. Since miRNAs play key roles in tumor development and have a high specificity and low immunogenicity, miRNA-target delivery strategies have shown promise for anti-tumor therapy. Chen et al. [33] showed that reconstituted high-density lipoprotein-nanoparticles (rHDL-NPs) could effectively deliver miR-204-5p inhibitor (miR-204-5p-inh) to tumor sites and inhibit tumor growth by upregulating the expression of THBS1. Our results showed that miR-582-5p might account for the loss of INPP5B in LUAD patients. In light of this finding, there is a promising possibility of managing LUAD by modulating miR-582-5p/INPP5B/AKT axis.

## Conclusions

In conclusion, INPP5B, as a tumor suppressor gene, has the potential to be used as a novel biomarker in LUAD patients, which may shed new light on LUAD prevention and treatment in the future.

## Supplementary Information

**Additional file 1:** Supplementary Materials and Methods.

**Additional file 2:** Source data.

**Additional file 3: Table S1.** The statistics of patient information in the TCGA database. **Table S2.** The statistics of patient information in the immunohistochemistry slides.

**Additional file 4:** Figure S1.

## Data Availability

The datasets generated and/or analyzed during the current study are available in the (TCGA) repository (https://tcgadata.nci.nih.gov/tcga/) [34] and (GEO) repository (http://www.ncbi.nlm.nih.gov/geo) [35]. The datasets used and/or analyzed during the current study are available from the corresponding author on reasonable request.
