# Supplementary material for: Decreased INPP5B expression predicts poor prognosis in lung adenocarcinoma
Source: Cancer Cell Int. 2022 May 14;22:189. doi: 10.1186/s12935-022-02609-8 (PMC9107680; doi:10.1186/s12935-022-02609-8)
Supplement: Supplementary file 1 — Additional file 1: Supplementary Materials and Methods. [file 12935_2022_2609_MOESM1_ESM.doc]

**Additional methods**

**INPP5B expression analysis**

Level 3 RNA-sequencing data of LUAD patients from TCGA and corresponding normal tissue data in GTEx were extracted to analyze the differential expression of INPP5B. The GSE72094 dataset was used to validate. The RNAseq data in TPM (Transcripts per million reads) format were log_2_-transformed. The R programming language (version 3.6.3) and the R package “ggplot2” (version 3.3.3) were used for statistical analysis and visualization. The Wilcoxon signed-rank test was used to make statistical comparisons. (**p* < 0.05, ***p* < 0.01, and ****p* < 0.001)

The TNMplot online database (https://tnmplot.com/analysis/) was used to analyze the mRNA expression of INPP5B in pan-cancer, which includes 56,938 unique multilevel quality-controlled samples.

The UALCAN portal (http://ualcan.path.uab.edu/analysis-prot.html) is a web-based tool for conducting INPP5B protein expression analysis of the clinical proteomic tumor analysis consortium (CPTAC).

The HPA database (https://www.proteinatlas.org/), a web-based resource for analyzing cancer Omics data, enabled us to conduct a search for INPP5B expression and distribution in LUAD patients and normal lung tissues.

**The correlation between** **INPP5B** **expression and** **clinicopathological features of** **LUAD patients**

TCGA data with clinical information recorded was conducted to explore correlations between INPP5B mRNA expression and clinicopathological features in LUAD patients. The RNAseq data in FPKM (Fregments Per Kilobase per Million) format were log_2_-transformed. The R programming language (version 3.6.3) and the R package “ggplot2” (version 3.3.3) were used for statistical analysis and visualization. The Wilcoxon signed-rank test was used to make statistical comparisons.(**p* < 0.05, ***p* < 0.01, and ****p* < 0.001)

The OCLR method developed by Malta et al. was used to compute the stemness index. Based on the mRNA expression signature, the gene expression profile contains 11,774 genes. We used the same Spearman correlation (RNA expression data). The minimum value was subtracted, and the result was divided by the maximum maps the stemness index to the range [0,1].

The data of the three gene expression LUAD subtypes - terminal respiratory unit (TRU), proximal proliferative (PP), and proximal Inflammatory (PI) - were collected from the TCGA Portal. (http://www.tcgaportal.org/TCGA/Lung_TCGA_LUAD/index.html)

The Normal, Tumor and Metastatic analysis page of the TNMplot online database (https://tnmplot.com/analysis/) provides detailed analysis for a selected gene in a selected tissue type using gene chip based data.

**ROC curve analysis**

By using the R package “pROC(version 1.17.0.1)”, the diagnostic utility of INPP5B in LUAD patients was evaluated by receptor operating characteristic (ROC) curve analysis based on GTEx normal lung samples, TCGA LUAD samples, and GSE10072 datasets. The abscissa is the False Positive Rate (FPR), and the ordinate is the True Positive Rate (TPR). The area under the ROC curve (AUC) was used to analyze the performance of ROC curve. From these ROC curves, an area under the curve (AUC) value of 0.5-0.7 represented low accuracy, 0.7-0.9 represented high accuracy, and 0.9-1.0 represented extremely high accuracy.

**Survival prognosis analysis**

The R packages “survival” (version 3.2-10) and “survminer” (version 0.4.9) were used to conduct survival and cox analyses based on TCGA data. Statistics were calculated by log-rank test using the Mantal-Cox method. The surv-cutpoint function from the R package survminer was used to determine the optimal cutpoint for separating continuous variables. In the univariate analysis, all variables with *p-*value less than 0.1 were included in the multivariate analysis.

On the basis of GeneChip data, the Kaplan-Meier plotter online database (http://kmplot.com/analysis/) was used to assess the prognostic potential of a selected gene in LUAD. The examination probe ID of INPP5B was 220580_at. By selecting the "Restrict analysis to treatment groups" option, the relationship between INPP5B expression and clinical treatment benefit rate was explored. By selecting the "auto select best cut-off" option, all possible cut-off values were computed and the threshold with the best performance was used as the cut-off.

**The gene co-expression analysis**

The co-expressed genes of INPP5B were calculated by the R(version 3.6.3) package “stat” based on the TCGA project's expression datasets of the LUAD transcriptome. Results were presented using the R package “ggplot2”(version 3.3.3). Additionally, the co-expressed genes with INPP5B was validated in GEPIA browser (http://gepia.cancer-pku.cn/index.html)[33]. The statistical significance of correlations was assessed with Spearman’s rank correlation coefficient test. *P* < 0.05 were considered statistically significant.

**Biological functional** **analysis**

Using the R package "ClusterProfiler"(version 3.14.3), gene enrichment analysis of Gene Ontology (GO) and Kyoto Encyclopedia of Genes Genome (KEGG) were performed on co-expressed genes of INPP5B with Spearman rho greater than 0.30. Pathways enriched were considered significant with *p*.adjust < 0.05 and *q* value < 0.2.

In Gene set enrichment analysis (GSEA) analysis, we first generated an ordered list of all genes based on their correlation with INPP5B expression as the method described above. Default settings and the MSigDB C2 curated KEGG gene sets (c2.cp.v7.2.symbols.gmt) were used for the analysis. Each analysis included a 1000 times permutation of the gene set. Pathways enriched with False discovery rate(FDR) < 0.25 and *p*.adjust < 0.05 were considered to be significant. Results were presented using the R package “ggplot2”(version 3.3.3).

**Protein-protein interaction analysis**

GeneMANIA (http://www.genemania.org)[31] and STRING database (https://string-db.org/)[32] were used to probe the INPP5B-involved protein-protein interaction (PPI) network. After combining the results of GeneMANIA and STRING, we selected overlapping genes as hub genes.

**Mutation analysis**

The TCGA mutation and expression data were obtained from cBioportal (http://www.cbioportal.org). Default settings and the TCGA PanCancer Atlas dataset were used for the analysis. The OncoPrinter (version 1.0.1) tools of cBioPortal were used to produce the mutation graphs.

**Methylation analysis**

SurvivalMeth (http://bio-bigdata.hrbmu.edu.cn/survivalmeth/) is, a database to study the impact of DNA methylation-related functional elements on prognosis, allowed us to investigate the association between methylation and expression of INPP5B, as well as the relationship between methylation and survival prognosis of INPP5B.

**Targeted miRNA Prediction**

PicTar (2007), PITA (version catalog version 6), and miRmap (miRmap-1.1.tar.gz) databases were used for exploring miRNA targeting INPP5B. After combining the results of the above databases, we selected the overlapping one and predicted its binding site with INPP5B. In addition, we analyzed the correlation between the miRNA and INPP5B expression in the TCGA database and analyzed the expression of the miRNA in LUAD patients and the impact on patient survival as the method described above.

**Immunohistochemistry (IHC) analysis**

Briefly, 4-μm sections from paraffin-embedded tumor tissues were deparaffinized in xylene and dehydrated in graded alcohols. For antigen retrieval, the slides were boiled in 10 mM citrate buffer in a microwave for 20 min and then cooled at room temperature for 30 min. Endogenous peroxidase activity was quenched by incubating the slides in 0.3% H_2_O_2_ and then washing with phosphate-buffered saline (PBS). The slides were incubated with the anti-INPP5B primary antibody (proteintech, 15141-1-AP, 1:200). After washing with PBS, the slides were incubated with the secondary antibody in a humidified chamber for 60 min, followed by 3,3-diaminobenzidine (DAB) chromogen and haematoxylin nuclear counterstaining. Positive control tissue was stained in parallel. The intensity of staining was defined by H-score: H-score = (weak intensity% × 1) + (moderate intensity% × 2) + (strong intensity% × 3).

**CCK8 assay**

Cell viability was determined using the Cell Counting Kit-8 (CCK8) assay (MedChem Express, USA). Following transfection, cells were seeded at a density of 500 cells per well in 96-well plates; absorbance values were determined 0 to 96 hours after transfection.100 µL RPMI 1640 medium solution containing 10 μL of CCK8 was added to each well of the 96-well plates and incubated for an additional 2 hours. The absorbance at 450 nm was then determined using a microplate reader (Bio-Rad, USA). Each experiment had three duplicate wells and was performed three times.

**Colony formation assay**

Following the transfection, cells were counted and cultured in six-well plates with culture media at a density of 500 cells per well. Every three days, the medium of cultivation was changed. Colonies were counted only if they contained at least 50 cells. The cells were stained with 0.5% crystal violet for 30 minutes at room temperature, washed five times with tap water, allowed to air dry and then photographed and counted. Each experiment was carried out three times in all.

**Cell cycle assay**

Cells were seeded on 60 mm dishes at a density of 1 × 10^6^. Transfection was carried out after starvation in serum-free media for 12 h. Cells were harvested 48 h post-transfection for flow cytometry analysis. For cell cycle analysis, cells were collected and then digested using trypsin with 0.25% EDTA. After centrifugation to remove supernatant, the cells were washed three times with PBS, followed by the addition of 70% chilled ethanol and overnight storage at 4°C; the cells were then washed again, centrifuged after resuspension, and cycle detection reagent (550825, BD, USA) was added for 30 minutes. Following incubation, samples were examined by flow cytometrically (BD Biosciences) and the results were processed using ModFit LT 2.0. The experiment was repeated three times independently.

**Wound healing assay**

Cells were seeded on 6-well plates at a density of 9 × 10^5^. A micropipette tip was used to scrape the cell monolayer at 100% confluence 24 hours after transfection. After washing twice with PBS, serum-free medium (inhibiting the cell proliferation) was added. Photographs were obtained at 0h and 24h post-scratch with a 10× optical microscope (Nikon, Ti-E, Japan). The scratch area was calculated to obtain the migration percentage. Cell migration was calculated using the formula: % Cell Migration=100% × (original scratch width- final scratch width) / (original scratch width). The experiment was repeated three times independently .

**Transwell migration assay**

After 24 hours of transfection, serum-free medium was used to prepare cell suspensions. The upper chamber of a 24-well plate was supplemented with 200 μLof 1640 medium containing 5 × 10^4^ cells, and the lower chamber was supplemented with 800 μL of 1640 medium containing 20% FBS. After 24 hours of incubation at 37 °C, cells were washed twice with PBS solution, fixed for 20 minutes at room temperature with methanol, stained with 0.5% crystal violet solution, and observed under a 10× optical microscope (Nikon, Ti-E, Japan). Each experiment was conducted a minimum of three times.

**Immunoprecipitation**

The cells were lysed with IP buffer and the lysates were centrifuged at 10,000 g for 30 min at 4°C. The cell lysates were incubated with protein G magnetic beads for 1 h at 4°C. The subsequent immunoprecipitation reactions were set up with equal quantities of the lysates (no-antibody, nonreactive antibody-Mouse IgG, and specific antibody). The no-antibody lysates were added into the loading buffer. The other lysates were added 10 μL protein G magnetic beads then rotated overnight at 4°C by tightly securing the caps. The beads were washed with wash buffer at least five times, mixed with loading buffer, and then heated to 95°C for 5 min, followed by immunoblotting.
