# Supplementary material for: Decreased INPP5B expression predicts poor prognosis in lung adenocarcinoma
Source: Cancer Cell Int. 2022 May 14;22:189. doi: 10.1186/s12935-022-02609-8 (PMC9107680; doi:10.1186/s12935-022-02609-8)
Supplement: Supplementary file 3 — Additional file 3: Table S1. The statistics of patient information in the TCGA database. Table S2. The statistics of patient information in the immunohistochemistry slides. [file 12935_2022_2609_MOESM3_ESM.doc]

**Table S1. The statistics of patient information in the TCGA database**.

| Characteristic | Low expression of INPP5B | High expression of INPP5B | p |
| --- | --- | --- | --- |
| n | 267 | 268 |  |
| Gender, n (%) |  |  | 0.008 |
| Female | 127 (23.7%) | 159 (29.7%) |  |
| Male | 140 (26.2%) | 109 (20.4%) |  |
| Age, n (%) |  |  | 1.000 |
| <=65 | 127 (24.6%) | 128 (24.8%) |  |
| >65 | 129 (25%) | 132 (25.6%) |  |
| Pathologic stage, n (%) |  |  | < 0.001 |
| Stage I | 128 (24.3%) | 166 (31.5%) |  |
| Stage II | 67 (12.7%) | 56 (10.6%) |  |
| Stage III | 57 (10.8%) | 27 (5.1%) |  |
| Stage IV | 11 (2.1%) | 15 (2.8%) |  |
| T stage, n (%) |  |  | 0.079 |
| T1 | 78 (14.7%) | 97 (18.2%) |  |
| T2 | 147 (27.6%) | 142 (26.7%) |  |
| T3 | 32 (6%) | 17 (3.2%) |  |
| T4 | 10 (1.9%) | 9 (1.7%) |  |
| N stage, n (%) |  |  | 0.004 |
| N0 | 158 (30.4%) | 190 (36.6%) |  |
| N1 | 53 (10.2%) | 42 (8.1%) |  |
| N2 | 49 (9.4%) | 25 (4.8%) |  |
| N3 | 1 (0.2%) | 1 (0.2%) |  |
| M stage, n (%) |  |  | 0.398 |
| M0 | 198 (51.3%) | 163 (42.2%) |  |
| M1 | 11 (2.8%) | 14 (3.6%) |  |
| Primary therapy outcome, n (%) |  |  | < 0.001 |
| PD | 50 (11.2%) | 21 (4.7%) |  |
| SD | 18 (4%) | 19 (4.3%) |  |
| PR | 4 (0.9%) | 2 (0.4%) |  |
| CR | 148 (33.2%) | 184 (41.3%) |  |
| Smoker, n (%) |  |  | 0.013 |
| No | 26 (5%) | 49 (9.4%) |  |
| Yes | 227 (43.6%) | 219 (42%) |  |
| Age, meidan (IQR) | 66 (59, 72) | 66 (59, 73) | 0.449 |

**Table S2. The statistics of patient information in the immunohistochemistry slides**.

| Clinical characteristics | Total | % |
| --- | --- | --- |
| Age at diagnosis, years |  |  |
| <65 | 3 | 30 |
| >65 | 7 | 70 |
| Sex |  |  |
| Male | 3 | 30 |
| Female | 7 | 70 |
| Stage |  |  |
| I | 7 | 70 |
| II | 1 | 10 |
| III | 2 | 2 |
| IV | 0 | 0 |
| T stage |  |  |
| T1 | 5 | 50 |
| T2 | 4 | 40 |
| T3 | 1 | 10 |
| T4 | 0 | 0 |
| N stage |  |  |
| N0 | 8 | 80 |
| N1 | 0 | 0 |
| N2 | 2 | 20 |
| N3 | 0 | 0 |
| M stage |  |  |
| M0 | 10 | 100 |
| M1 | 0 | 0 |
| Mx | 0 | 0 |
