# Supplementary figures and images for: Decreased INPP5B expression predicts poor prognosis in lung adenocarcinoma
Source: Cancer Cell Int. 2022 May 14;22:189. doi: 10.1186/s12935-022-02609-8 (PMC9107680; doi:10.1186/s12935-022-02609-8)

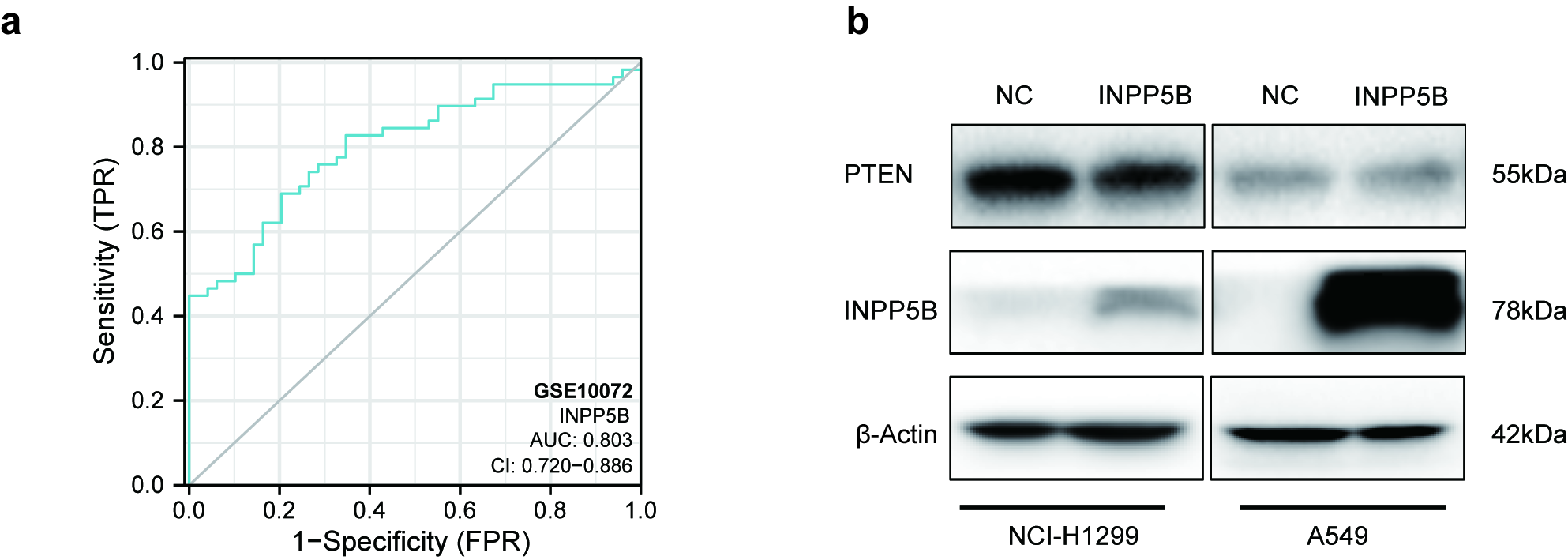

Supplement: Supplementary file 4 — Additional file 4: Figure S1. [file 12935_2022_2609_MOESM4_ESM.tif]
